# Optimising Nursing Management: Development of a Tool to Determine Span of Control and Resource Needs of First-Line Nurse Managers in Spanish Hospitals—A Mixed-Methods Study

**DOI:** 10.3390/healthcare13172215

**Published:** 2025-09-04

**Authors:** Ángel Boned-Galán, Nieves López-Ibort, Ana I. Gil-Lacruz, Carmen Angustias Gómez-Baca, Ana Gascón-Catalán

**Affiliations:** 1 Clinical Neurophysiology Unit, Miguel Servet University Hospital, 50009 Zaragoza, Spain; acboned@salud.aragon.es; 2 Aragon Health Research Institute (IIS Aragon), 50009 Zaragoza, Spain; nlopezi@salud.aragon.es (N.L.-I.); anagil@unizar.es (A.I.G.-L.); cagomez@salud.aragon.es (C.A.G.-B.); 3 Home Dialysis Unit, Lozano Blesa University Clinical Hospital, 50009 Zaragoza, Spain; 4 Department of Business Management and Organization, School of Engineering and Architecture, University of Zaragoza, 50018 Zaragoza, Spain; 5 Intensive Care Unit, Lozano Blesa University Clinical Hospital, 50009 Zaragoza, Spain; 6 Department of Physiatry and Nursing, Faculty of Health Sciences, University of Zaragoza, 50009 Zaragoza, Spain

**Keywords:** nursing, span of control, nurse manager, leadership, hospital, management, organisation, health services management

## Abstract

**Background**: First-Line Nurse Managers (FLNMs) have been recognised as key contributors to achieving organisational objectives, serving as vital intermediaries between management, staff, and patients. Assessing whether the Span of Control (SOC) is appropriate and providing the necessary support for FLNMs to fulfil their responsibilities poses a considerable challenge for healthcare organisations. No tool exists in Spain to guide decisions regarding FLNM’s SOC and resource needs. The aim of this study is to design a tool for assessing the span of control of first-line nurse managers in hospitals. **Methods**: This study employed a tool development and content validation design to create the EASOC-Nursing instrument (Eliges Aragón SOC tool). The study was conducted in three stages: an integrative literature review, followed by a national Delphi study with 43 experts in nursing management, and finally, focus group discussions. **Results**: A tool was created to assess first-line nurse managers’ (FLNM) span of control (SOC) using 13 key indicators, organised into four categories: unit (operations and resources, conflicts, and logging and monitoring of activities), professional (staff and competencies), FLNM (autonomy, education, and leadership), and organisation (support systems, education, and research). It includes a total of 31 items and determines SOC adequacy by establishing cut-off points that classify it as below acceptable, appropriate, or excessive. When the SOC is inadequate, the tool provides specific recommendations for support measures, such as the provision of administrative personnel or the appointment of a co-leader. **Conclusions**: The EASOC-Nursing tool offers a comprehensive evaluation of the core dimensions of the FLNM role and its responsibilities in Spain. Furthermore, it delivers practical guidance on the most suitable types of support to facilitate the attainment of optimal outcomes for both patients and healthcare organisations. In light of the global nursing shortage, the availability of a robust and context-sensitive instrument to assess the SOC enables hospital nursing management to allocate resources more strategically, thereby enhancing working conditions for professionals and contributing to improved patient care outcomes.

## 1. Introduction

The complexity of healthcare is increasing due to a number of factors, including advances in therapies and technologies, the need to provide person-centred care, the aging of the population, and the emergence of new pandemics. It is important to note that the majority of direct interventions on patients and/or caregivers are carried out by nursing staff (approximately 70%) and that they consume at least 65% of the budget set aside for human resources in a hospital. Therefore, it is essential to focus on areas that allow for improvements in the management of nursing units [1].

Nurse managers are instrumental in fulfilling the objectives of healthcare organisations within their respective nursing units and departments [2,3]. This is particularly evident in areas concerning the leadership and coordination of quality improvement initiatives [4]. The nurse manager collective includes first-line nurse managers (FLNMs).

The role of first-line nurse managers is characterised by their direct accountability for patient or resident care units, with staff nurses and other care personnel reporting directly to them. They are entrusted with responsibilities such as recruitment and performance management. Notably, there are no subordinate management levels beneath them, and they may be tasked with overseeing multiple units [5].

The administration of human resources is one of the core responsibilities of FLNMs. This is where span-of-control (SOC) theory becomes relevant. The term “SOC” was first used in business in the 1930s to refer to the quantity of employees under the responsibility of a supervisor [6]. But, as our understanding of the topic has grown, the term’s meaning has evolved, going from referring to the number of full-time equivalent employees [7] to the number of individuals who report to a manager [8].

According to span of control theory, there is a SOC size at which maximal efficacy can be attained. Despite this, there is no perfect figure or formula in the literature for figuring out how many direct reports there should be within an ideal span of control [9].

A recent review of the literature indicates that SOC constitutes a multidimensional phenomenon, shaped by a range of factors associated with the workplace, unit personnel, FLNM, and the organisation [7,10,11,12,13,14,15].

The selection of a specific SOC is contingent upon the organisational structure in relation to the groups and human resources to be managed by nursing management, as well as the number of FLNMs that will be required within the organisation [16,17,18]. In most health systems, the units responsible for the FLNM have been established in accordance with the number of employees and resources to be managed, without consideration of the specific needs of the nursing units and services. This practise has the potential to result in the creation of inappropriate SOCs, which can have a detrimental impact on a number of different areas: patients, healthcare professionals, FLNM and organisations.

A limited number of studies have specifically analysed the impact of SOC and its consequences. These include studies that have addressed safety issues and decreased patient satisfaction [5,19,20], safety, and employee behaviours [21,22,23,24].

In 2022, the Canadian Nursing Advisory Committee recommended the SOC to be evaluated in order to ensure its suitability for FLNM. This would enable them to fulfil their assigned roles and be present to meet the needs of nurses and patients [25].

It is only in Canada and the United States that a tool has been implemented to determine the FLNMs’ SOC [26]. The tool, which has been designed in accordance with the three dimensions of unit, staff, and programme, comprises 8 indicators and 16 items in total.

It is important to recognise that the health systems of different countries are not uniform, and this has an impact on the functioning of health organisations. Consequently, it is imperative to be cognisant of the distinctive attributes of each health system to ensure the utilisation of SOC measurement tools that are congruent with the prevailing circumstances. Given the absence of a suitable tool adapted to the Spanish public health context for the objective description and measurement of factors influencing FLNMs’ functions and responsibilities, we undertook the development of one.

Both FLNMs and organisational planning decision-makers highlight the need for practical, validated tools that are sensitive to the clinical context. Such instruments are essential to ensure the equitable and transparent allocation of workload, to identify risks associated with an imbalanced SOC, and to support structural decision-making processes at the managerial level based on robust scientific evidence [24].

The lack of a standardised instrument generates considerable variability in workload, allocation of responsibilities, and the number of professionals or units under each FLNM’s supervision. Without uniform criteria for assigning responsibilities, some FLNMs may become overburdened while others operate within a more restricted scope, resulting in internal inequities and an increased risk of professional burnout [27].

To address the issue of the lack of valid instruments for assessing SOC, a multifaceted approach is required, which encompasses both the development of measurement tools and the implementation of organisational and leadership strategies [24,28].

The findings reported by the American Organization for Nursing Leadership (AONL) in their analysis of the SOC in hospitals suggest that a broader SOC imposes a significantly greater workload, resulting in extended working hours [29]. As the average SOC increases, there is a corresponding rise in the number of hours devoted to managerial responsibilities each week.

One of the main consequences of an excessive and inadequate SOC is position abandonment. In 2022, the AONL found that 45% of Nurse Managers (NMs) were considering leaving their positions, with professional burnout and lack of work–life balance identified as the primary contributing factors [30]. In Spain, although precise figures are not available, a useful point of reference is the survey conducted by the Ministry of Health in 2024, which indicates that 39.4% of nurses are considering leaving the profession within the next ten years [31].

The development of the tool presented in this study is grounded in key principles of organisational management and nursing leadership, notably the SOC theory [6], which posits that supervisors can manage an optimal number of subordinates by considering factors such as task complexity, staff competence, and organisational context. Within the nursing domain, this aligns with transformational leadership frameworks that emphasise team empowerment, adaptive leadership, and patient safety—dimensions that are inherently influenced by SOC [32]. The purpose of this tool is twofold: firstly, to determine the SOC, and secondly, to propose the most appropriate type of support for the FLNM. The design process not only considers the number of professionals but also other aspects related to patients, the FLNM, and the organisation.

This document outlines the methodology employed in the development of a SOC assessment tool, including the challenges encountered, the processes undertaken, and recommendations for future tool development.

The main aim of this study is to develop a tool for assessing the span of control (SOC) of first-line nurse managers (FLNMs) in hospital healthcare centres.

## 2. Materials and Methods

The methods utilised to develop the tool that would capture the SOC of FLNMs included a literature review, a national Delphi study, a focus group, and preparation of the tool (Table 1). The first two methodological phases—the literature review and the Delphi study—have been previously published by our team and provide the empirical and expert foundation on which the tool was designed [10,33]. A summary of the methodology used in these studies is presented below, and the complete development process is described later in this section. This study was approved by Research Ethics Committee of the Autonomous Community of Aragon: CEICA (C.P.–C.I. PI22/351). Informed consent to participate was obtained from all of the participants in this study.

### 2.1. Literature Review: Identification of Concept, Problem Formulation, and Theoretical Framework

A comprehensive review of the literature was undertaken utilising Medline, Web of Science, and Embase, with pertinent articles identified through the application of the following search terms: “nurse administrator”, “nurse manager”, “first line nurse manager”, “nursing supervisor”, “head nurse”, “nurse management”, “charge nurse”, “span of control”, “span of management”, “work group size”, “and nursing”. The theoretical framework for this study is grounded in organisational contingency theory and nursing management theory. It posits that the SOC of FLNMs is not a fixed metric but rather a contingent variable determined by a combination of interrelated factors: unit characteristics, team composition, leader attributes, and the organisational context.

Our research question was as follows: Among first-line nurse managers (FLNMs), does the application of a multifactorial analytical framework—incorporating structural unit characteristics, professional team composition, leadership attributes, and organisational context—facilitate the identification of key determinants of the span of control?

An initial search yielded a total of 62 articles across 3 databases. After duplicate removal and screening of titles and abstracts based on predefined inclusion criteria, 29 studies were selected for full-text review. The quality of each publication was assessed using the Johns Hopkins Evidence-Based Practice Model to determine levels of evidence and quality [34]. In total, 21 articles met the inclusion criteria and were included in the final synthesis.

The findings of the review indicated that SOC is a multifaceted concept that is influenced by and has an effect on patients, healthcare professionals, the FLNM itself, and the organisation. Details of the literature review process and its results have been previously published and are available in reference [10].

### 2.2. Delphi Study Item Generation

To assist in the design of a tool and to determinate the elements that should be included in a SOC tool, a national Delphi study was conducted. The results of this study were previously published by our research team in reference [33]. The study involved 43 participants, including nurse administrators, FLNMs, university professors, renowned researchers, and other non-health professionals related to health management. The participants undertook an online survey conducted across three distinct phases, with the objective of identifying the principal determinants of SOC of FLNMs in hospitals. The number of respondents in each phase was as follows: 29 in phase 0, 31 in phase 1, and 29 in phase 2. A total of 22 participants completed all three phases of the survey.

In the first phase of the Delphi study, participants were asked to answer the following question: “List the most important factors to consider when determining the span of control of a nurse manager”. In order to prevent any undue influence on the input of the expert panel, only the item “Number of employees in charge of the FLNM” was provided as an example.

Afterwards, the qualitative responses from Phase 0 were subsequently transcribed and subjected to a thematic analysis to identify recurring themes. This analysis was conducted independently by the researcher and the Delphi study coordinating group. The concepts generated in the preceding phase were then used to formulate the items for the subsequent second and third rounds of questionnaires.

These themes constituted the basis of an initial questionnaire, organised into various dimensions, for evaluation by a panel of experts. For this purpose, a five-point Likert scale was employed to respond to the questions, without differentiating between positive and negative impacts on SOC. The questions pertained to two dimensions: (a) the extent to which the item was considered relevant to the management of FLNM’s SOC; and (b) whether the item ought to be incorporated into a definitive SOC assessment instrument for FLNMs. Consensus was established according to two criteria: (I) over 80% of participants in the round were required to indicate “YES” regarding the necessity of including the item in a tool for determining the SOC of an FLNM; and (II) the item needed to achieve a mean score of three or higher on the five-point scale.

Finally, the study showed a total of 31 items which should be taken into account to determine the SOC of a FLNM, revealing very different scopes of responsibility. Additional details, as well as a more comprehensive Delphi study that was conducted, are available in reference [33].

Once the 31 items that would comprise the tool had been agreed upon, the detailed drafting of each of the indicators was carried out. In order to achieve this objective, the following actions were undertaken:

- Definition of the items, the levels of each of them, and the weight they would have within the SOC of a FLNM. For example, in the case of the item “Staff volume”, the graduation was made according to the following scale: Low = Level 1 (less than 30 employees), Medium = Level 2 (31 to 70 employees), Medium-high = Level 3 (71 to 100 employees), High = Level 4 (more than 100 employees). This item has a weight of 5 points to be multiplied by the corresponding level.
- Weighting of the type of support recommended. The most appropriate type of support was established according to the criteria of suitability, competencies required to develop the functions, and efficient use of economic resources. Continuing with the previous example, Level 1 = no support required, Level 2 = adds 1 point for administrative support, Level 3 = adds 1 point for administrative support and 2 for clinical co-leader, Level 4 = adds 1 point for administrative support and 3 points to indicate the need for a second FLNM to head the unit.

### 2.3. Testing the Tool with FLNMs

To assess the reliability of the tool, a field test was conducted involving 10 FLNMs from 2 tertiary level hospitals. In each hospital, five FLNMs were paired with their counterparts, who were responsible for managing the same units. Participants were selected based on their representativeness regarding the SOC within their respective centres, considering factors such as the number of units managed, staffing levels, work environment, and absenteeism. Additionally, four FLNMs from one of the hospitals served as reference cases (a medical hospitalisation unit, a surgical unit, a unit where different types of therapies are performed, and a unit without direct patient care).

After being informed of the purpose of the study, participants received an email containing instructions on how to complete the attached file, which included the tool developed using Microsoft Excel. The participants were asked to select the most appropriate option for their specific context in relation to each of the 31 items comprising the tool. To prevent bias, neither the item weightings nor the final score were disclosed at this stage. These were provided only after the completed questionnaire had been received.

### 2.4. Focus Group with FLNMs: Content Validity Assessment and Pilot Testing

Subsequently, a focus group was conducted, to which all 10 FLNMs were invited in order to provide feedback on the relevance, clarity, and weighting of the items, at the facilities of the University of Zaragoza, a public institution, close to their workplaces and outside of working hours, thereby ensuring a neutral environment for the participants. It was proposed to review the understanding and weighting of the various items and to discuss possible modifications.

The participants selected for the focus group were FLNMs with more than two years of experience in nursing management. In addition, they were chosen because they were leading units that cared for the same type of patients (emergency, onco-haematology, dialysis, internal medicine, and cardiology). One FLNM was selected from each unit and each hospital in order to form representative pairs from both centres. This methodological step contributes to enhancing the content validity of the tool. While the context may influence some aspects of external validity, the participation of experienced FLNMs from different units provides relevant insights applicable to similar settings.

Seven participants attended, while the remaining three were unable to participate due to scheduling conflicts or personal circumstances. The contributions of the focus group made it possible to redefine some of the items to ensure better understanding (either through rewording or by adding illustrative examples) and to clarify the weighting of the types of support required. The COREQ guidelines were followed in reporting the study process [35]. This manuscript was drafted against the Consolidated Criteria for Reporting Qualitative Research (COREQ): for qualitative research. (https://www.equator-network.org/reporting-guidelines/coreq/ (accessed on 22 November 2024)).

The modifications and examples proposed for the wording of the items enhanced the clarity of the statements and helped to prevent potential misinterpretations. The suggestions made regarding the weightings provided a more realistic and contextually appropriate perspective aligned with the group’s overall situation.

Once the proposed modifications had been made, the final questionnaire and the SOC assessment using the tool were sent to each of the participants. It was unanimously agreed that the results reflected the SOC, adequately differentiated the various areas of responsibility, and allowed the most appropriate type of support to be defined. The instrument demonstrated an acceptable level of internal consistency, with a Cronbach’s alpha of 0.756, indicating satisfactory reliability.

## 3. Results

As a result of the process mentioned above, the tool Eliges’ Aragón Span of Control –Nursing—(EASOC-Nursing) (available as Supplementary File_EASOC Nursing tool) was created, which includes 4 decision-making categories to classify 13 indicators for a total of 31 items. The four categories were unit, professional, FLNM, and organisation.

The unit-focused category includes 4 indicators: 1—complexity of the unit (characteristics of the unit and its operation as well as of the patients and the relationships established between them to ensure continuity of care), 2—resource management (differentiating between consumables, drugs, and management of equipment and facilities), 3—conflicts and complaints (related to patients and professionals in the unit), and 4—protocolisation and monitoring of activities (degree of standardisation in the activities carried out in the unit, monitoring of objectives and incidents/accidents affecting patients and/or workers).

Three indicators are outlined under the professional-focused category: 1—volume of staff directly reporting to the FLNM (making reference to all professionals and not only to full-time employees), 2—staffing stability and skill level (percentage of junior professionals without the necessary skills to perform their functions in a fully autonomous manner, turnover of professionals, and absenteeism rate), and 3—diversity of staff (healthcare or non-healthcare personnel who depend on the FLNM to perform their functions even if the FLNM is not their hierarchical superior).

The FLNM-focused category includes three indicators: 1—autonomy (refers to the ability to make decisions that affect the unit without requiring authorisation from a superior), 2—experience and education (includes years of experience in different management positions and training), and 3—leadership style (distinguishing between positive leadership styles such as transformational, transactional, and the rest). The education item, made up of 5 sub-items (differentiating between masters or higher education, management training, soft skills, digital skills, and nursing methodology), is the only one in the entire questionnaire that contributes a negative value in the weighting of the FLNMs’ SOC.

**Figure 1:**
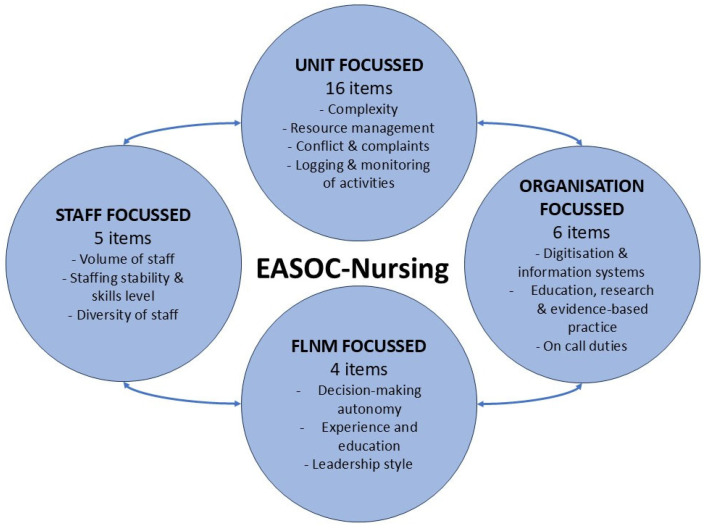
Categories, indicators, and number of items in the EASOC-Nursing tool.

The final category, organisation-focused, has three indicators: 1—digitisation and information systems (availability of IT solutions that provide information and facilitate work), 2—education, research, and implementation of evidence-based practise (actions related to the acquisition of skills and knowledge of students and professionals, research, and the existence of stable groups or commissions that provide support), and 3—on call-duty (responsible for different units during weekends and holidays). Figure 1 shows the indicators and the total number of items in each category.

### Focus Group Characteristics

For the revision of the weighting and wording of the items in the EASOC-Nursing tool, we collaborated with a focus group. Ten FLNMs were invited to participate, with seven participants, of whom six were women and one was a man. A total of 57.1% were responsible for two or more non-contiguous care units, 71.4% had more than 10 years of management experience, and only one of them was responsible for more than 100 professionals. The main characteristics can be seen in Table 2.

All of the participants stated that they agreed with the items included in the tool. In some cases, it was necessary to modify the wording of the items to avoid ambiguities. For example, in item P01: Staff size, it was necessary to add the following text: “Refers to the total number of professionals, not to the core staff. Take into account the percentage of workday reductions, slippage, and other types of leave for work-life balance”. Another modified item was U10, which was edited to include the completion and follow-up of facility repair reports.

**Figure 2:**
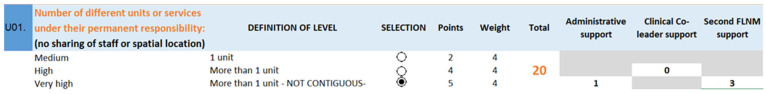
Example weighting matrix for a single item in the EASOC-Nursing tool. Each item has a singular weight that multiplies the score assigned based on the FLNM’s span of control. In this example, being responsible for more than 1 non-contiguous unit scores 5 points, which multiplied by the singular weight results in 20 points for the overall span of control (SOC) score. Relative to this score, one would score 1 point for the final administrative support score and 3 points for the recommendation to assign a second FLNM. The same procedure is applied to all 31 items, each of which has specific scoring criteria based on different levels. For further information, please refer to Supplementary File S1: EASOC Nursing Tool.

Subsequently, the weighting of the items and the types of support recommended was evaluated. A total of 100% of the participants agreed with the proposed scoring and weighting of the items. If it was necessary to modify the type of support recommended or the weighting of items U01, U02, U10, U15, F01, and F04, the new values were agreed upon by the focus group. Figure 2 shows the weighting matrix for one of them, illustrating how scores are assigned based on different response options.

To apply the tool, the value of each item is calculated by multiplying the points assigned to the item by its respective weight (Figure 2). To assess whether the FLNM requires additional support, the total score obtained across all items related to the specific type of support must be calculated. If the cumulative score exceeds 50% of the maximum possible score for that type of support—namely, administrative support (9 points), clinical co-leader (26 points), or an additional FLNM (13 points)—it is recommended that the organisation evaluate the need to provide such support. For further information, please refer to Supplementary File: EASOC Nursing Tool.

After carrying out the focus group, the cut-off points that refer to the SOC were established. For this purpose, the scores obtained by administering the final version of the EASOC-Nursing tool were reviewed and discussed independently with each of the focus group participants. Finally, the SOC was divided into 3 levels: 1—SOC < 50%, span of control is below acceptable, capable of growth; 2—SOC > 50% (112 points) and <60% (135 points), appropriate SOC; and 3—SOC > 60%, excessive SOC, FLNM requires support.

## 4. Discussion

The present study outlines the development and face validation process of a new tool for the evaluation of the SOC of FLNM tailored to the Spanish public healthcare system. To the best of our knowledge, this is the first tool developed and adapted for this context, although other tools, such as the TOH-SOC [26], have been employed by various authors [5,19,28,36,37]. However, these tools are oriented towards different healthcare systems that differ significantly from our own. For instance, the inclusion of indicators related to unit budget management—an area that does not fall within the remit of FLNMs in the Spanish public health system—illustrates this misalignment. Until now, the implementation of SOC-related policies has encountered several challenges. These include practical contingencies, a lack of empirical studies, definitional ambiguities, and the emergence of new organisational models and technologies [38].

A total of 11 items from the TOH-SOC were used in the development of the EASOC-Nursing tool, maintaining those related to professionals (5 items), part of those referring to the unit (5 items), and 1 related to the program. Nevertheless, it was necessary to incorporate additional items, as agreed upon by the expert panel during the Delphi study, in order to complete the unit dimension and to establish the FLNM and organisational domains.

In the design of the new items, one notable outcome we would like to highlight is the inclusion of aspects related to the FLNM in determining their SOC. In addition to the traditional item—such as years of management experience—new items have been incorporated, including autonomy in decision-making, educational background, and leadership style.

Undoubtedly, the primary role of the FLNMs is to ensure the effective functioning of the unit. They act as catalysts for operationalising the organisation’s mission, vision, and values, empowering staff and striving to achieve optimal patient outcomes. Autonomy in decision-making can enhance work efficiency and effectiveness by reducing delays and eliminating unnecessary intermediary steps. It also contributes to motivation and talent retention by making staff feel more valued [39,40]. However, this autonomy can also impose an additional workload on the FLNMs, a factor that must be carefully considered when evaluating their overall responsibilities. For this reason, a high level of autonomy has been assigned the recommendation for clinical co-leader support.

Another key aspect influencing the performance of FLNMs is their education, both prior to assuming the role and throughout their professional careers [15]. This body of knowledge constitutes the foundation of the tools they require to effectively lead the unit or units under their responsibility. In the present study, both postgraduate education (at the master’s level) and other types of knowledge—such as soft skills [41,42], nursing methodology [43], digital competencies [44,45], and leadership abilities [15,28]—have been considered separately. These are the only items in the EASOC-Nursing tool that carry a negative weighting, meaning that they deduct points from the FLNM’s SOC score. This is because the greater the knowledge and competencies of the FLNM, the better equipped they are to manage the complexities of unit leadership and team coordination.

Leadership style holds particular relevance in relation to SOC when guiding professional teams. Several authors highlight the importance of adopting positive leadership styles, with transformational leadership being particularly effective in healthcare environments [46,47,48]. This leadership style emphasises inspiring and motivating staff to reach their full potential and has been shown to enhance job satisfaction, employee engagement, and performance. However, “there is no leadership style capable of overcoming the effects of a large SOC” [8], and this has significant implications for professional satisfaction [49], patient outcomes [20,49], and staff turnover or intent to leave [20].

Using positive leadership styles will allow FLNMs to lead the professionals within their units more effectively. However, in order to implement these styles, it is essential not only to possess the requisite skills and knowledge but also to establish effective communication, promote collaboration and teamwork, act as ethical and professional role models, and foster practices that enhance connection and meaning at work. Nonetheless, adopting and sustaining these behaviours demands a significant investment of time and effort from FLNMs, thereby increasing their workload and responsibilities.

FLNM can enhance nurses’ professional autonomy through shared leadership. However, disparities remain in nurses’ ability to exert equal influence within multiprofessional teams, particularly beyond direct patient care [50]. Promoting autonomy requires commitment and support from leadership at all levels of the organisation. The findings recommend that both FLNMs and organisational leaders maximise the potential of nurses’ expertise while also encouraging the development of self-leadership among nursing staff.

FLNMs typically dedicate a substantial proportion of their time to administrative tasks [51]. Implementing appropriate technological solutions can not only enhance efficiency and productivity in managing human and material resources but also yield benefits in other critical areas. These include improving training and professional development of the entire care team, facilitating the evaluation and monitoring of clinical practice, optimising communication, and ensuring the availability of relevant indicators in a timely and accessible manner to support informed decision-making.

Among the items grouped under education, research and evidence-based practise are some of the most relevant, but often neglected, functions that FLNMs should perform [26,52,53]. This oversight often occurs because more pressing responsibilities, such as managing human and material resources, consume the majority of their time. As the leaders of their units, FLNMs must ensure that both healthcare professionals and trainees acquire the necessary knowledge and competencies, always grounded in the best available evidence. Their role is not to deliver training directly, but to assess levels of competence, identify areas for improvement, and ensure that appropriate actions are implemented. These functions should be supported by quality units, working groups, and clinical care committees. When such groups are in place, their work may be leveraged by FLNMs to enhance clinical workflows and the quality of patient care within their units, thereby enabling them to dedicate more time to other essential managerial duties.

Similarly, involvement in research and the implementation of evidence-based best practises demands a considerable investment of time and effort from FLNMs. Their commitment to these activities can significantly contribute to the prestige of the unit and the healthcare organisation as a whole. While FLNMs are not necessarily expected to lead these initiatives—particularly when qualified professionals are available—they should remain informed and supportive of the research and evidence-based activities being conducted within their teams.

On the other hand, the fact that FLNMs are required to be on duty for the entire organisation, outside their units, usually on weekends and holidays, acting as representatives of the nursing management of the centre to solve possible incidents, is a stressful situation and an extra workload. This responsibility, which they carry out only occasionally, involves managing potentially complex situations in units and services with which they may be largely unfamiliar. Such tasks further increase the demands placed on FLNMs, often without corresponding adjustments to their primary responsibilities.

A key challenge in assessing SOC has been the determination of a cut-off point to determine whether the level is appropriate. Although the literature recognises it as a multifactorial concept, the majority of the studies reviewed adhere to a classical definition, focusing exclusively on the number of subordinates assigned to a supervisor [54]. In our study, three levels were established using the TOH-SOC tool as a reference [26] and were adapted following field testing conducted with FLNMs who participated in a focus group. It was therefore determined that when the overall SOC score exceeds 60%, the FLNM is experiencing a higher-than-recommended level of demand and thus requires additional support.

In other words, when the overall SOC score is above 60%, or when the organisation considers it appropriate—even if the score is slightly lower—it is time to consider support-type indicators (administrative, clinical co-leader, or second FLNM). A cut-off point of 50% has been established for these three indicators, and it is possible for more than one to exceed this threshold. In such cases, the nursing management of the institution must take into account both the objectives to be achieved and the economic context of the organisation (e.g., enhancing safety, fostering professional development, improving management processes, and containing costs). These factors will determine the prioritisation of certain units over others, as well as the type of support to be provided.

One of the novelties provided by the EASOC-Nursing tool is its ability to recommend the type of support required by the FLNM. This support may be purely administrative, involve the appointment of a clinical co-leader, or, in some cases, necessitate the collaboration of a second FLNM to assume the responsibilities originally assigned to a single individual. There is precedent for this approach: providing administrative support has been shown to reduce the workload of FLNMs, enabling them to devote more time to staff training and to increase their presence in clinical areas [28]. Along the same lines, the Michigan Leadership Model [55] highlights the benefits of additional support for FLNMs, incorporating both administrative and leadership support positions on an as-needed basis. An integrative review on time allocation [51] further revealed that one of the most commonly expressed needs by FLNMs themselves was for greater administrative support. In our study, although administrative support has been considered—and has been reported as effective in several studies [28,56]—it has been assigned limited importance. This is due to the fact that in our healthcare system, it is not always feasible to have professionals in this category who possess the necessary training, experience, job stability, capability, and/or motivation to carry out the tasks that may be delegated to them by the FLNM. Furthermore, historically, only the medical profession and high-level management positions have benefited from such support, typically following a formal selection process to assess candidates’ suitability for the role—something that is generally not feasible in other cases.

Our main approach, coinciding with the available evidence and the contributions of the focus group, has been to support the FLNM with a clinical co-leader. This support allows the FLNM to focus on management tasks that require the authority of the role, delegating complex tasks related to clinical practise to the clinical co-leader.

This is a figure who must possess a combination of skills, including a strong specialised clinical background in the area in which they work, critical thinking abilities, leadership, communication, and interprofessional collaboration [57,58,59]. Their responsibilities may include tutoring, mentoring, and training newly arrived professionals in the unit, tailoring support to the individual needs of each person in order to more effectively enhance the skills and competencies of staff [60]. The clinical co-leader must be granted the necessary recognition and authority to prevent issues concerning the legitimacy of the role [22]. With the support of a clinical co-leader, in those units with excessive SOC, it will be possible to improve the quality of care, foster the professional development within the team, and optimise decision-making. Such collaboration contributes to a more efficient, satisfying, and patient-centred working environment.

However, it will not always be possible to mitigate excessive SOC through administrative support or a clinical co-leader. In certain situations, the demands placed on the FLNM are so considerable that they warrant dividing the role between two individuals with equal authority. Key factors that support this recommendation include being responsible for two or more non-contiguous units, overseeing a workforce of more than 100 professionals, or managing units with high rates of absenteeism. In such cases, appointing an additional FLNM to share the workload and responsibilities may be justified. This approach may help to reduce SOC and, as evidenced by Cathcart’s research [21], improve unit outcomes, enhance employee engagement, and positively influence other nursing behaviours [61].

The evaluation of the FLNM’s SOC should be a continuous and adaptive process, adjusting to the needs and changes of the organisation [28,52]. Periodic review, along with the consideration of specific circumstances and the integration of autonomous teams, are recommended practices. Based on our experience, it would be advisable to administer it at least at three key moments: a few months after the FLNM assumes the position (recommended approximately two months) [62]; at least every 4 years (to observe potential changes in the professionals, the unit, the FLNM, and the organisation); and every time important changes occur within the unit or the organisation that justify re-evaluation.

### 4.1. Limitations

One of the limitations of the tool is that the initial categorisation of the items and the assignment of weights, both to measure SOC and the type of support, was done taking into account the experience of the research team. To improve accuracy, a preliminary test was performed and agreed upon with a group of experts, adjusting a small number of them. We acknowledge that this study represents a preliminary phase in the development of the instrument, limited to face and content validity assessment. Therefore, further studies are needed to conduct more rigorous psychometric testing, including factor analysis and reliability assessments, to confirm the structural validity and consistency of the instrument. It should also be tested in other centres and health systems with similar characteristics.

As the tool is mainly oriented to assess the SOC of FLNMs at the head of care units, it is possible that its applicability may not be as effective in other types of units. Likewise, it may not be adapted to specific situations and extreme cases; for example, in a pandemic situation.

### 4.2. Implications for Nursing Management

This study offers significant implications for nursing management, extending beyond FLNMs to encompass patients, professionals, and the organisation. This study provides a tool to objectively and comprehensively measure the SOC of FLNMs. This information can be used to develop interventions for decision making regarding FLNM support and the allocation of resources, whether human, material, or technological, as well as to analyse the results of the improvement actions implemented. Along the same lines, the results obtained after analysing the SOC will allow for inter-comparability between different units and/or organisations.

The implementation of the tool is particularly relevant in the Spanish healthcare system, where nursing shortages and difficulties in staff retention, especially among FLNMs, are critical challenges. Improving the conditions under which FLNMs perform their roles may contribute to strengthening leadership, enhancing staff wellbeing, and ultimately improving the quality and safety of patient care. Beyond the individual unit, the tool also supports nursing directors in aligning resource allocation with organisational objectives, offering a framework for comparability between different hospitals or services. Although designed for the Spanish public hospital context, the tool can also be adapted to private institutions or other healthcare systems with similar organisational characteristics, thus expanding its applicability and contributing to international research on SOC in nursing management.

## 5. Conclusions

The importance of the SOC of FLNMs to the results for patients, professionals, the FLNM themselves, and the organisation has been demonstrated in multiple studies. Consequently, it is essential to evaluate it periodically using tools that consider factors beyond the mere number of employees or units under the FLNM’s supervision. This newly developed tool, EASOC-Nursing, offers a comprehensive assessment of the key dimensions that define the role and responsibilities of FLNMs. Furthermore, it enables the calculation of whether the size of the SOC is appropriate for a given unit and the characteristics of the FLNM, as well as identifying the necessary supports, either administrative or professional, with the role of a co-leader or FLMN, required for the optimal functioning of the unit.

Once it has been demonstrated that the size of the SOC is inappropriate, taking into account the characteristics of the unit, the professionals, the organisation, and the FLNM itself, it is possible to make informed decisions that support the need to provide support. This will allow resources to be allocated to those people or units that most need them, in accordance with the objectives pursued by the organisation.

Given the global nursing shortage, the EASOC-Nursing tool holds significant international relevance. Although developed for the Spanish public healthcare system, its methodology can serve as a model for other nations seeking to create context-specific instruments for strategic resource allocation. This approach ensures that staffing policies go beyond simple headcount, providing a framework for healthcare organisations.

While this study establishes the development and face validity of the EASOC-Nursing tool, future research will focus on strengthening its psychometric properties. The next logical step is a large-scale validation study to assess its reliability and construct validity in a diverse sample of first-line nurse managers across Spain. The long-term implementation of the tool will also allow for the assessment of its predictive validity, analysing correlations between scores and outcomes like staff turnover or patient care quality. Ultimately, international validation will underscore the tool’s global applicability.

## Figures and Tables

**Table 1:** Development process of the EASOC-Nursing tool.

Phase	Purpose	Method	Results
Phase 1	To describe the available scientific knowledge regarding FLNM’s SOC in hospitals.	Integrative literature review of peer-reviewed publications.	A total of 21 full-text articles were included. The SOC was found to influence patients, professionals, FLNMs, and healthcare organisations.
Phase 2	To identify and reach consensus on the key aspects related to the SOC of FLNM in public healthcare system hospitals in Spain.	National three-round Delphi study including nurse administrators, FLNM, university professors, renowned researchers, and other non-health professionals related to health management.	A total of 31 items related to the FLNMs’ SOC were identified and validated through expert consensus
Phase 3	To develop and conduct face validation of the EASOC-Nursing tool to assess the FLNM’s SOC in Spanish public healthcare system hospitals.	Classification, stratification, weighting, and preliminary estimation of the type of support suggested for the 31 items. Pilot test involving 10 FLNMs from 2 tertiary hospitals. Focus group conducted with 7 of the 10 FLNMs from the pilot study to assess statement clarity, weighting, and the suggested type of support.	The EASOC-Nursing tool was developed, comprising 4 categories (unit, professionals, FLNM, and organisation), 13 dimensions, and 31 items.

**Table 2:** Sociodemographic and professional characteristics of focus group members involved in the EASOC-Nursing tool review.

	N	%
Sex		
Female	6	85.71
Male	1	14.29
Experience (years in management)		
From 2 to 10 years	2	28.57
More than 10 years	5	71.42
Number of units under your responsibility		
1 unit	1	14.29
More than 1 unit	2	28.57
More than 1 non-contiguous	4	57.14
Number of professionals in charge		
From 51 to 70	2	28.57
From 71 to 100	4	57.14
More than 100	1	14.29
Education		
Master’s	2	28.57
Management	3	42.86
Soft Skills	6	85.71
Digital skills	7	100
Nursing methodology	4	57.14
Leadership style		
Transformational	3	42.86
Transactional	4	57.14

## Data Availability

The data used in this study are available upon reasonable request to the corresponding author, subject to any ethical and legal constraints.

## Supplementary Materials

The following supporting information can be downloaded at: https://www.mdpi.com/article/10.3390/healthcare13172215/s1, Supplementary File: EASOC Nursing tool.

## Abbreviations

The following abbreviations are used in this manuscript:

AONL	American Organization for Nursing Leadership
EASOC-Nursing tool	Eliges Aragon Span of Control Nursing tool
FLNM	First-Line Nurse Managers
SOC	Span of Control
TOH-SOC	The Ottawa Hospital Clinical Managers Decision-Making Tool

## References

[1] Salvadores P., Sanchez F., Jimenez R. (2002). Manual de Administración de Los Servicios de Enfermería.

[2] Krugman M., Smith V. (2003). Charge Nurse Leadership Development and Evaluation. JONA J. Nurs. Adm..

[3] Shuman C.J., Ploutz-Snyder R.J., Titler M.G. (2018). Development and Testing of the Nurse Manager EBP Competency Scale. West. J. Nurs. Res..

[4] McKimm J., Till A. (2015). Clinical Leadership Effectiveness, Change and Complexity. Br. J. Hosp. Med..

[5] Wong C., Elliott-Miller P., Laschinger H., Cuddihy M., Meyer R.M., Keatings M., Burnett C., Szudy N. (2015). Examining the Relationships between Span of Control and Manager Job and Unit Performance Outcomes. J. Nurs. Manag..

[6] Urwick L.F.V.A. (1974). Graicunas and the Span of Control. Acad. Manag. J..

[7] Altaffer A. (1998). First-Line Managers. Measuring Their Span of Control. Nurs. Manag..

[8] Doran D., Mccutcheon A., Evans M., Macmillan K., Hall L.M., Pringle D., Smith S., Valente A. (2004). Impact of the Manager’s Span of Control on Leadership and Performance.

[9] Meier K.J., Bohte J. (2000). Ode to Luther Gulick: Span of Control and Organizational Performance. Adm. Soc..

[10] Boned-Galán A., López-Ibort N., Gascón-Catalán A. (2023). Nurse Manager Span of Control in Hospital Settings: An Integrative Review. Nurs. Rep..

[11] Hattrup G., Kleiner B. (1993). How to Establish the Proper Span of Control for Managers. Ind. Manag..

[12] Meyer R.M. (2008). Span of Management: Concept Analysis. J. Adv. Nurs..

[13] OHA (2011). Leading Practices for Addressing Clinical Manager Span of Control in Ontario.

[14] Pabst M.K. (1993). Span of Control on Nursing Inpatient Units. Nurs. Econ..

[15] Ruffin A., Shirey M.R., Dick T., Fazeli P.L., Patrician P.A. (2023). Understanding the Impact of Span of Control on Nurse Managers and Hospital Outcomes. J. Healthc. Manag..

[16] Stoner J., Whankel C. (1986). Management.

[17] Koontz H., O’ Donnell C. (1974). Essentials of Management.

[18] Asaria M., McGuire A., Street A. (2022). The Impact of Management on Hospital Performance. Fisc. Stud..

[19] Cupit T., Stout-Aguilar J., Cannon L., Norton J. (2019). Assessing the Nurse Manager’s Span of Control: A Partnership Between Executive Leadership, Nurse Scientists and Clinicians. Nurse Lead..

[20] McCutcheon A., Doran D., Evans M., Hall L., Pringle D. (2009). Effects of Leadership and Span of Control on Nurses’ Job Satisfaction and Patient Satisfaction. Nurs. Leadersh..

[21] Cathcart D., Jeska S., Karnas J., Miller S.E., Pechacek J., Rheault L. (2004). Span of Control Matters. JONA J. Nurs. Adm..

[22] Holm-Petersen C., Østergaard S., Andersen P.B.N. (2017). Size Does Matter—Span of Control in Hospitals. J. Health Organ. Manag..

[23] Lee H., Cummings G.G. (2008). Factors Influencing Job Satisfaction of Front Line Nurse Managers: A Systematic Review. J. Nurs. Manag..

[24] Omery A., Crawford C.L., Dechairo-Marino A., Quaye B.S., Finkelstein J. (2019). Reexamining Nurse Manager Span of Control With a 21st-Century Lens. Nurs. Adm. Q..

[25] Nursing Advisory Committee Our Health, Our Future: Creating Quality Workplaces for Canadian Nurses. https://oaresource.library.carleton.ca/cprn/30762_en.pdf.

[26] Morash R., Brintnell J., Rodger G. (2005). A Span of Control Tool for Clinical Managers. Nurs. Leadersh..

[27] Shirey M.R., Ebright P.R., McDaniel A.M. (2013). Nurse Manager Cognitive Decision-Making amidst Stress and Work Complexity. J. Nurs. Manag..

[28] Simpson B.B., Dearmon V., Graves R. (2017). Mitigating the Impact of Nurse Manager Large Spans of Control. Nurs. Adm. Q..

[29] AONL Analyzing Span of Control for Frontline Clinical Managers at General Hospital. https://www.aonl.org/system/files/media/file/2024/06/Example_Span-of-Control%20Report_Custom_Template.pdf.

[30] AONL AONL Foundation 2022 Longitudinal Nursing Leadership Insight Study. https://www.aonl.org/system/files/media/file/2022/10/AONL%20Longitudinal%20Nursing%20Leadership%20Insight%20Study%20Report%20L4%20Final.pdf.

[31] Ministerio de Sanidad Situación Actual y Estimación de La Necesidad de Enfermeras En España. https://www.sanidad.gob.es/areas/cuidadosEnSalud/investigacionDatos/docs/SITUACION_ACTUAL_Y_ESTIMACION_DE_LA_NECESIDAD_DE_ENFERMERAS_EN_ESPANA_2024_PPT.pdf.

[32] Boamah S.A., Spence Laschinger H.K., Wong C., Clarke S. (2018). Effect of Transformational Leadership on Job Satisfaction and Patient Safety Outcomes. Nurs. Outlook.

[33] Boned-Galán A., López-Ibort N., Gil-Lacruz A.I., Gascón-Catalán A. (2024). Determinants of First-Line Nurse Managers’ Span of Control: A Delphi Study. J. Nurs. Manag..

[34] Dearholt S., Dang D., Bissett K., Ascenzi J., Whalen M. (2012). Johns Hopkins Nursing Evidence Based Practice Model and Guidelines.

[35] Tong A., Sainsbury P., Craig J. (2007). Consolidated Criteria for Reporting Qualitative Research (COREQ): A 32-Item Checklist for Interviews and Focus Groups. Int. J. Qual. Health Care.

[36] Jones D., McLaughlin M., Gebbens C., Terhorst L. (2015). Utilizing a Scope and Span of Control Tool to Measure Workload and Determine Supporting Resources for Nurse Managers. J. Nurs. Adm..

[37] Merrill K., Pepper G., Blegen M. (2013). Managerial Span of Control: A Pilot Study Comparing Departmental Complexity and Number of Direct Reports. Nurs. Leadersh..

[38] Zoller Y.J., Muldoon J. (2020). Journey of a Concept: Span of Control—The Rise, the Decline, and What Is Next?. J. Manag. Hist..

[39] Fallman S.L., Jutengren G., Dellve L. (2019). The Impact of Restricted Decision-making Autonomy on Health Care Managers’ Health and Work Performance. J. Nurs. Manag..

[40] Krairiksh M., Anthony M.K. (2001). Benefits and Outcomes of Staff Nurses’ Participation in Decision Making. JONA J. Nurs. Adm..

[41] Agomoh C.J., Brisbois M.D., Chin E. (2020). A Mapping Review of Clinical Nurse Leader and Nurse Educator Transitional Care Skills and Competencies. Nurs. Outlook.

[42] Bundy H., Sunkara P., Sitammagari K., Hetherington T., Hole C., Murphy S. (2024). Soft Skills: The Work of Communication and Persuasion Among Nurse Navigators in Hospital at Home Programs. JONA J. Nurs. Adm..

[43] González García A., Pinto-Carral A., Pérez González S., Marqués-Sánchez P. (2022). A Competency Model for Nurse Executives. Int. J. Nurs. Pract..

[44] Collins S., Yen P.-Y., Phillips A., Kennedy M.K. (2017). Nursing Informatics Competency Assessment for the Nurse Leader. JONA J. Nurs. Adm..

[45] Laukka E., Hammarén M., Pölkki T., Kanste O. (2023). Hospital Nurse Leaders’ Experiences with Digital Technologies: A Qualitative Descriptive Study. J. Adv. Nurs..

[46] Al-Hussami M., Hamad S., Darawad M., Maharmeh M. (2017). The Effects of Leadership Competencies and Quality of Work on the Perceived Readiness for Organizational Change among Nurse Managers. Leadersh. Health Serv..

[47] Palweni V.S., Malesela J.M., Randa M.B. (2023). Nurse Managers’ Leadership Styles as an Impetus to Patient Safety in an Academic Hospital. Health SA Gesondheid.

[48] Suliman M., Aljezawi M., Almansi S., Musa A., Alazam M., Ta’an W.F. (2020). Effect of Nurse Managers’ Leadership Styles on Predicted Nurse Turnover. Nurs. Manag..

[49] Jankelová N., Joniaková Z. (2021). Communication Skills and Transformational Leadership Style of First-Line Nurse Managers in Relation to Job Satisfaction of Nurses and Moderators of This Relationship. Healthcare.

[50] Pursio K., Kankkunen P., Kvist T. (2023). Nurse Managers’ Perceptions of Nurses’ Professional Autonomy—A Qualitative Interview Study. J. Adv. Nurs..

[51] Bjerregård Madsen J., Kaila A., Vehviläinen-Julkunen K., Miettinen M. (2016). Time Allocation and Temporal Focus in Nursing Management: An Integrative Review. J. Nurs. Manag..

[52] Wong C., Elliott-Miller P., Laschinger H., Cuddihy M., Meyer R., Keatings M., Burnett C., Szudy N. (2014). Exploring Managers’ Views on Span of Control: More Than a Headcount. Can. J. Nurs. Leadersh..

[53] Clavijo-Chamorro M.Z., Romero-Zarallo G., Gómez-Luque A., López-Espuela F., Sanz-Martos S., López-Medina I.M. (2022). Leadership as a Facilitator of Evidence Implementation by Nurse Managers: A Metasynthesis. West. J. Nurs. Res..

[54] Urwick L. (1956). The Manager’s Span of Control. Harv. Bus. Rev..

[55] Dawson C., Aebersold M., Mamolen N., Goldberg J., Frank C. (2005). The Michigan Leadership Model: Developing a Management Infrastructure. J. Nurs. Adm..

[56] Pegram A.M., Grainger M., Sigsworth J., While A.E. (2014). Strengthening the Role of the Ward Manager: A Review of the Literature. J. Nurs. Manag..

[57] Bender M., Connelly C.D., Brown C. (2013). Interdisciplinary Collaboration: The Role of the Clinical Nurse Leader. J. Nurs. Manag..

[58] Huber D. (2018). Leadership and Nursing Care Management.

[59] Meyer R.M., O’Brien-Pallas L., Doran D., Streiner D., Ferguson-Paré M., Duffield C. (2011). Front-Line Managers as Boundary Spanners: Effects of Span and Time on Nurse Supervision Satisfaction. J. Nurs. Manag..

[60] De Vries N., Lavreysen O., Boone A., Bouman J., Szemik S., Baranski K., Godderis L., De Winter P. (2023). Retaining Healthcare Workers: A Systematic Review of Strategies for Sustaining Power in the Workplace. Healthcare.

[61] Ibort N.L., Galán A.B., Lairla M.C., Lombarte T.A., Catalán A.G. (2021). Impact of Charge Nurses’ Span of Control on the Work Attitudes of Nurses. An. Sist. Sanit. Navar..

[62] Liden R., Sparrowe R., Wayne S. (1997). Leader-Member Exchange Theory: The Past and Potential for the Future. Res. Pers. Hum. Resour. Manag..

