# Supplementary material for: Optimising Nursing Management: Development of a Tool to Determine Span of Control and Resource Needs of First-Line Nurse Managers in Spanish Hospitals—A Mixed-Methods Study
Source: Healthcare (Basel). 2025 Sep 4;13(17):2215. doi: 10.3390/healthcare13172215 (PMC12427949; doi:10.3390/healthcare13172215)
Supplement: Supplementary file 1 [file healthcare-13-02215-s001.zip › healthcare-3827268-supplementary.pdf]

ELIGES' SPAN OF CONTROL -NURSING-(EASOC-NURSING) TOOL

1. INSTRUCTIONS.. 1
2. QUESTIONNAIRE... 1
3. WEIGHTING TABLE 8
4. INTERPRETATION OF RESULTS 11

1. INSTRUCTIONS

Please select the value for each of the 31 items in the questionnaire based on the corresponding definitions. Subsequently, multiply the points for each item by its respective weighting factor.

In a subsequent phase, sum the points corresponding to the suggested type of support. To accomplish this, utilise the values provided in the weighting table section.

Then add up all of the totals for each item and the suggested type of support. Finally place your grand total on section interpretation of results.

Example:

U01.	Number of different units or services under their permanent responsibility: (no sharing of staff or spatial location)	DEFINITION OF LEVEL	SELECTION	Points	Weight	Total	Administrative support	Clinical Co-leader support	Second FLNM support
	Medium	1 unit	○	2	4				
	High	More than 1 unit	○	4	4	20		0	
	Very high	More than 1 unit - NOT CONTIGUOUS-	◉	5	4		1		3

2. QUESTIONNAIRE

UNIT / SERVICE RELATED ITEMS

INDICATORS ON UNIT COMPLEXITY

ID	ITEM DEFINITION	DEFINITION OF LEVEL	POINTS	WEIGHT	TOTAL
**U01**	**Number of different units or services under their permanent responsibility:** (no sharing of staff or spatial location)				
	Medium	1 unit	2	4	
	High	More than 1 unit	4	4	
	Very high	More than 1 unit - NOT CONTIGUOUS-	5	4	
**U02**	**Variability / complexity of patient care:** *- Number of different care processes / medical specialties attended in the care unit. Consideration will be given to those units with a high percentage of ectopic patients, understood as those patients who require care different from what the unit staff is used to (for inpatient units).*				
	Low	Units with little variability and/or no direct patient care.	1	3	
	Medium	Moderate variability in type of patients and care required. Ectopic patients only occasionally. Less than 3 medical specialties in the unit.	2	3	
	High	High variability in the type of patients and the care they require, and frequent presence of ectopic patients. Existence of more than three medical specialties in the care unit.	3	3	
**U03**	**Hours of operation of the unit / service:**				
	Low	Weekdays only 8 – 15.	1	2	
	Medium	Extended hours (morning and afternoon shift	2	2	
	High	24/7 (services avaliable including standby).	3	2	

**U04**	**Unpredictability of the work of the unit or service**:			
	Low	Never or rarely (at most once a week it is necessary to reassign personnel on a shift).	1	3
	Medium	Occasionally (2 to 5 times a week it is necessary to reassign/ rellocate staff on a shift).	2	3
	High	Frequently (more than 5 times a week it is necessary to reassign/ rellocate staff on a shift).	3	3
**U05**	**Frequency with which the capacity of the unit or service is exceeded:**<br>*This refers to patients located within the unit itself. For example, patients located in the emergency room pending admission to the inpatient unit when beds are available will not be taken into account.*			
	Low	Never or hardly ever (maximum once a week the capacity of the unit/service is exceeded).	1	2
	Medium	Occasionally (2 to 5 times a week the capacity of the unit/service is exceeded).	2	2
	High	Frequent (more than 5 times a week the capacity of the unit/service is exceeded.	3	2
**U06**	**Interdisciplinary Communication and the Maintenance of Care Processes and Circuits:**<br>*- Number of medical professionals and/or other specialties that interrelate in the unit, making it necessary to coordinate the different services involved.*<br>*Unit scheduling management. It will refer to units where a high number of tests are performed on patients that need to be coordinated, surgical units....*			
	Low	Patients are routinely attended to by the staff of the unit (for all professional categories) or by staff assigned to the unit or service (e.g., physiotherapists in the Stroke Unit). Collaboration with other units or services is only requested on a one-off basis. Patients undergo a low number of examinations outside the unit.<br>This section includes diagnostic units, outpatient clinics, surgical block, major outpatient surgery	1	2
	Medium	**Usually,** less than 25% of patients are seen by professionals from other units or services. Moderate number of procedures performed outside the unit or service.<br>Surgical inpatient units will be included.	2	2
	High	**At** least 25% of the patients are attended daily by professionals who do not belong to the unit or service. Performance of a large number of diagnostic tests.<br>This section includes emergencies and weekday hospitalization.	3	2
**U07**	**Number of patients attended in the units/services under the responsibility of the FLNM:**			
	No patient care	Services or units without patient care	0	2
	Low	Services or units where patients attend an ad hoc basis by appointment (radiology, outpatient services, laboratory for sampling, rehabilitation, etc.) or scheduled as chronic patients (haemodialysis, etc.).	1	2
	Medium	Inpatient units with LESS than 40 total beds.<br>Weekday and Day Hospital.	2	2
	High	Inpatient units with MORE than 40 total beds.<br>Emergency, surgical block, CMA, functional tests, haemodynamics ...	3	2
**U08**	**Rotation of patients in the unit or service:**			
	No patient rotation	Services or units without patient care	0	2
	Low	Services or units where patients attend on an ad hoc basis by appointment (radiology, outpatients ...) or scheduled as chronic patients (hemodialysis ...).	1	2
	Medium	Units where daily patient turnover is less than 20%.	2	2

	High	Units where daily patient turnover exceeds 20%.	3	2	

RESOURCE MANAGEMENT INDICATORS

ID	ITEM DEFINITION	DEFINITION OF LEVEL	POINTS	WEIGHT	TOTAL
**U09**	**Material resource management:** *Material not included in Kamban, smart cabinets or other automated management systems. No staff available to assist in these functions (nursing support workers, administrative staff).*				
	Low	< 4 hours/week.	1	2	
	Medium	4 - 8 hours/week.	2	2	
	High	> 8 hours/week.	3	2	
**U10**	**Management of a large volume of equipment/equipment/facility maintenance:** Must meet one or more of the following characteristics: high technology, needs to be used by several professionals at the same time, obsolescence and/or service-related problems, no technological solutions available to facilitate the management of this equipment, facility maintenance requests must be made by the FLNM).				
	Low	< 4 hours/week.	1	2	
	Medium	4 - 8 hours/week.	2	2	
	High	> 8 hours/week.	3	2	
**U11**	**Pharmacy Management:** Dedication to the replenishment of medicine cabinets, incidents with Unidosis or automatic dispensing systems such as PYXIS.<br>Special attention to units where chemotherapy drugs are administered, a large number of parenteral nutrition or other special medications such as narcotics carried out by the Pharmacy department.				
	Low	< 4 hours/week.	1	2	
	Medium	4 - 8 hours/week.	2	2	
	High	> 8 hours/week.	3	2	

INDICATORS ON CONFLICTS AND COMPLAINTS

ID	ITEM DEFINITION	DEFINITION OF LEVEL	POINTS	WEIGHT	TOTAL
**U12**	**Work climate of the Unit / Service:** *Commitment of the professionals to the organisation and/or time dedicated to conflict management and to maintaining an adequate work environment.*				
	Low	Units with a good working environment.	1	2	
	Medium	Units where the FLNM must intervene **OCCASIONALLY** to mediate disputes between workers.	2	2	
	High	Units where the FLNM must intervene **FREQUENTLY** (daily or almost daily) to mediate disputes between workers or where written submissions are made to the center's management.	3	2	
**U13**	**Communication with patients, relatives and/or companions**: *Attention to complaints made by patients, their relatives or accompanying persons, communicated directly or through the User Care Unit.*				
	Units without patient care	There are no patient complaints.	0	2	
	Low	Specific complaints.	1	2	
	Medium	Frequent complaints.	2	2	
	High	Daily complaints.	3	2	

INDICATORS ON LOGGING AND MONITORING OF ACTIVITIES

ID	ITEM DEFINITION	DEFINITION OF LEVEL	POINTS	WEIGHT	TOTAL
**U14**	**Degree of protocolisation of the unit or service:** *Existence of protocols and standardized care plans or systematized work procedures for routine activities.*				
	Low	Non-existent standardisation or only for some procedures.	3	2	
	Medium	LESS than 50% of activities are standardised.	2	2	

	High	MORE than 50% of activities are standardised.	1	2
**U15**	**Monitoring activities and achievement of unit or service objectives:** *(whether or not included in the program contract)*.			
	No follow-up	No follow-up of activities.	0	2
	Low	Dedication of LESS than 2 hours/week.	1	2
	Medium	Dedication 3-5 hours/week.	2	2
	High	Dedication MORE THAN 5 hours/week.	3	2
**U16**	**Dedication to risk management and** patient **safety:** *Safety culture, degree of implementation of safe practices in the unit / service.*			
	Low	Dedication of LESS than 2 hours/week.	1	4
	Medium	Dedication 3-5 hours/week.	2	4
	High	Dedication MORE THAN 5 hours/week.	3	4

ELIGES' SPAN OF CONTROL –NURSING- (EASOC-NURSING)
A tool for the assessment of the Span of control of First Line Nurse Managers

STAFFING RELATED ITEMS

STAFFING VOLUME INDICATOR

ID	ITEM DEFINITION	DEFINITION OF LEVEL	POINTS	WEIGHT	TOTAL
**S01**	**Staffing volume:** ***This refers to the total number of professionals, not to the core staff.*** *Take into account % of reduced working hours,slippage, and other types of leave for work-life balance.*				
	Low	< 30	1	5	
	Medium	31 - 70	2	5	
	Medium-high	71 - 100	3	5	
	High	> 100	4	5	

INDICATORS ON STABILITY AND SKILLS OF THE WORKFORCE

ID	ITEM DEFINITION	DEFINITION OF LEVEL	POINTS	WEIGHT	TOTAL
**S02**	**Number of novice professionals:** Includes professionals who have recently completed their studies, are working for the first time in a unit or service of these characteristics and/or do not have the necessary skills to perform most of their functions autonomously). ** It will be necessary to assess the need for job-oriented training of the professional (staff of the unit or service, but also "pool" staff).*				
	Low	< 5	1	3	
	Medium	5 - 15	2	3	
	High	> 15	3	3	
**S03**	**% Turnover of professionals in the unit (REGULAR AND INTERIM STAFF)**: Number of professionals on the staff who change each year. ** For all professional categories in the unit and for various reasons such as transfer competitions, internal mobility processes within the hospital, etc.*				
	Low	< 10	1	3	
	Medium	10 - 20	2	3	
	High	> 20	3	3	
**S04**	**Unit absenteeism:** % of absenteeism processes (monthly average) in relation to the total number of professionals in the unit. Occasioned for different reasons and for all the professionals in charge of the head of the nursing unit.				
	Low	< 5%	1	2	
	Medium	5% - 10%	2	2	
	High	> 10%	3	2	

WORKFORCE DIVERSITY INDICATOR

ID	ITEM DEFINITION	DEFINITION OF LEVEL	POINTS	WEIGHT	TOTAL
**S05**	**Number of different professional categories present in the unit under the responsibility of the FLNM:** Take into account other professionals who are not in charge of the unit but who are functionally and organisationally in charge of the unit (administrative, residents, cleaning staff, orderlies).				
	Low	1 - 3	1	2	
	Medium	4 - 6	2	2	
	High	> 6	3	2	

ELIGES' SPAN OF CONTROL –NURSING- (EASOC-NURSING)

A tool for the assessment of the Span of control of First Line Nurse Managers

FIRST LINE NURSE MANAGER RELATED ITEMS

INDICATOR ON DECISION-MAKING AUTONOMY

ID	ITEM DEFINITION	DEFINITION OF LEVEL	POINTS	WEIGHT	TOTAL
**F01**	**Decision-making autonomy**: *Related to all or some of the following aspects (HR management and recruitment, HR procurement, organisational aspects of her/his unit or service,...).*				
	Low	Must consult most of the aspects mentioned above.	1	3	
	Medium	Has a certain degree of freedom.	2	3	
	High	Fully autonomous within the framework of its competences.	3	3	

INDICATORS ON EXPERIENCE AND EDUCATION

ID	ITEM DEFINITION	DEFINITION OF LEVEL	POINTS	WEIGHT	TOTAL
**F02**	**Years of experience <u>IN MANAGEMENT</u>:** In charge of units/services or in other management functions				
	Low	< 2 years	3	3	
	Medium	2 - 10 years	2	3	
	High	> 10 years	1	3	
**F03**	**Education of the FLNM:** *This item is multiple choice. Select as many aspects as you consider relevant to fit your training*				
	01. Official university level	Master's Degree in Care Management and Direction, Master's Degree in Clinical Direction and Management...	1	-2	
	02. Other management training of more than 150 teaching hours	University expert degrees or the sum of different related training actions.	1	-2	
	03. Soft skills (overall training of more than 150 teaching hours)	Time management, effective communication, leadership, creativity and innovation...	1	-2	
	04. Digital competences and skills (formal training or self-taught)	Intermediate to advanced level office automation, mobile applications and online communication tools, audiovisual content creation...	1	-2	
	05. Nursing Methodology (over 150 hours)	Training in nursing methodology.	1	-2	

INDICATOR ON THE LEADERSHIP STYLE OF THE FLNM

ID	ITEM DEFINITION	DEFINITION OF LEVEL	POINTS	WEIGHT	TOTAL
**F04**	**Leadership style (according to the type of influence):**				
	Transformational leadership	The FLNM positively influences by encouraging professionals to step out of their comfort zone to perform at their best. It fosters growth and continuous improvement processes.	2	1	
	Transactional leadership	The FLNM recognises and rewards staff for a job well done through incentives* that serve to increase motivation and better define roles and responsibilities (*recognition, facilitating attendance at scientific activities or specific training...).	1	1	
	Other leadership styles	Any other leadership style that does not coincide with one of those mentioned above.	0	1	

ELIGES' SPAN OF CONTROL –NURSING- (EASOC-NURSING)
A tool for the assessment of the Span of control of First Line Nurse Managers

ORGANISATION RELATED ITEMS

INDICATOR ON DIGITISATION AND INFORMATION SYSTEMS

ID	ITEM DEFINITION	DEFINITION OF LEVEL	POINTS	WEIGHT	TOTAL
O01	**Digitalisation and technological support for HR and HRM management. Information systems:** Digital culture of the organisation; Automation of the management of the FLNM through ICTs (warehouse management, hospital discharges...); Existence of information systems that automate the management of payroll and inventory/ordering.				
	Low	**LOW** digititalisation of the institution. Existing applications tend to be obsolete and require a great deal of time on the part of the FLNM. Poor interconnection between applications, preventing or hindering data extraction.	3	3	
	Medium	**MODERATE** digitalisation of the institution. Only part of the processes are automated.	2	3	
	High	**HIGH** digitalisation of the institution. It uses digital tools, technologies and ecosystems that automate most of the work of the FLNM.	1	3	

INDICATORS ON EDUCATION, RESEARCH AND EVIDENCE-BASED PRACTICE

ID	ITEM DEFINITION	DEFINITION OF LEVEL	POINTS	WEIGHT	TOTAL
O02	**Evaluation of competencies and identification of training needs among unit professionals:** *particularly in relation to newly recruited staff and the implementation of new procedures, techniques, or work dynamics. This process includes the planning, execution, and evaluation of improvement initiatives. It does not necessarily imply that the entire process must be conducted by theFLNM.*				
	Low	Planning, implementation and evaluation of improvement actions related to competency assessment and training of professionals **RARELY FREQUENT.**	1	2	
	Medium	The planning, implementation and evaluation of improvement actions related to competence assessment and training of professionals **IS OCCASIONAL.**	2	2	
	High	The planning, implementation and evaluation of improvement actions **IS VERY FREQUENT.**	3	2	
O03	**Participation in research projects, EBP (Evidence Based Practice), R&D&I (Research, Development and Innovation) and dissemination of results:**				
	Low	There is no participation or only data collection.	1	2	
	Medium	The FLNM collaborates as a member of projects or groups.	2	2	
	High	The FLNM leads or actively participates in the coordination of one or more projects.	3	2	
O04	**Existence of trainees for tutoring in the unit / service:** *For all professional categories managed by the FLNM*				
	Low	Units with no students or where the FLNM has minimal contact with them.	1	2	
	Medium	The FLNM gives some seminars and monitors the evolution of the internships.	2	2	
	High	Existence of students in the last year of their training plan, with the FLNM being actively involved in the training. Units with specialist training nurses (e.g. midwifery students).	3	2	
O05	**Existence of cross-cutting units, commissions or working groups that provide support to the FLNM:** *Quality, patient safety, clinical care committees, etc.*				
	Low	The cross-cutting units and groups provide support to the FLNM on a regular basis.	1	2	
	Medium	Interaction with these units, committees or working Groups is occasional.	2	2	
	High	Most of these units do not exist or do not support the FLNM.	3	2	

INDICATOR ON THE PERFORMANCE OF ON-CALL DUTIES

ID	ITEM DEFINITION	DEFINITION OF LEVEL	POINTS	WEIGHT	TOTAL
**U06**	**Performance on-call duties as nurse in charge:**				
	Low	From 0 to 3 per year.	0	2	
	Medium	More than 4 on-call duty per year.	1	2	

3. WEIGHTING TABLE

UNIT / SERVICE RELATED ITEMS	Level	Points	Weight	Administrative support	Clinical co-leader support	second FLNM support
**U01. Number of different units or services under their permanent responsibility.**	Medium	2	4			
	High	4	4		2	
	Very high	5	4	1		3
**U02. Variability / complexity of patient care,**	Low	1	3			
	Medium	2	3		1	
	High	3	3		2	
**U03. Unit operating hours.**	Low	1	2			
	Medium	2	2			
	High	3	2		1	
**U04. Unpredictability of the work of the unit or service.**	Low	1	3			
	Medium	2	3			1
	High	3	3			2
**U05. Frequency with which the capacity of the unit or service is exceeded.**	Low	1	2			
	Medium	2	2			
	High	3	2		1	
**U06. Interdisciplinary communication and maintenance of processes and care circuits.**	Low	1	2			
	Medium	2	2		1	
	High	3	2		2	
**U07. Number of patients attended in the units/services under the responsibility of the FLNM**	No patient care	0	2			
	Low	1	2			
	Medium	2	2		1	
	High	3	2	1	2	
**U08. Rotation of patients in the unit or service.**	No patient care	0	2			
	Low	1	2			
	Medium	2	2			
	High	3	2		1	
**U09. Material resources management.**	Low	1	2			
	Medium	2	2			
	High	3	2	1		
**U10. Management of a large volume of equipment/equipment/facility maintenance.**	Low	1	2			
	Medium	2	2			
	High	3	2	1	1	
**U11. Pharmacy Management.**	Low	1	2			
	Medium	2	2			
	High	3	2		2	

**U12. Unit working climate.**	Low	**1**	**2**			
	Medium	**2**	**2**			
	High	**3**	**2**			**1**
**U13. Communication with patients, relatives and/or companions.**	There are no patient complaints	**0**	**2**			
	Low	**1**	**2**			
	Medium	**2**	**2**			
	High	**3**	**2**			**1**
**U14. Degree of protocolisation of the unit or service.**	Low	**3**	**2**		**2**	
	Medium	**2**	**2**		**1**	
	High	**1**	**2**			
**U15. Monitoring activities and achievement of unit or service objectives**	No follow-up	**0**	**2**			
	Low	**1**	**2**			
	Medium	**2**	**2**		**1**	
	High	**3**	**2**		**2**	
**U16. Dedication to risk management and patient safety.**	Low	**1**	**4**			
	Medium	**2**	**4**		**2**	
	High	**3**	**4**		**3**	

**If the type of support appears shaded in grey, it indicates that the level corresponding to that item has not been established for scoring.*

**STAFF RELATED ITEMS**	Level	Points	Weight	Administrative support	clinical co-leader support	second FLNM support
**S01. Staffing volume**	Low	**1**	**5**			
	Medium	**2**	**5**	**1**		
	Med -High	**3**	**5**	**1**	**2**	**2**
	High	**4**	**5**	**1**		**3**
**S02. Number of novice professionals**	Low	**1**	**3**			
	Medium	**2**	**3**		**1**	
	High	**3**	**3**		**2**	
**S03. % Turnover of professionals in the unit.**	Low	**1**	**3**			
	Medium	**2**	**3**		**1**	
	High	**3**	**3**		**2**	
**S04. Unit absenteeism.**	Low	**1**	**2**			
	Medium	**2**	**2**			
	High	**3**	**2**		**1**	**2**
**S05. Number of different professional categories present in the unit in charge of the FLNM.**	Low	**1**	**2**			
	Medium	**2**	**2**			
	High	**3**	**2**			**1**

ELIGES' SPAN OF CONTROL –NURSING- (EASOC-NURSING)
A tool for the assessment of the Span of control of First Line Nurse Managers

FLNM RELATED ITEMS	Level	Points	Weight	Administrative support	clinical co-leader support	second FLNM support
**F01. Decision-making autonomy**	Low	**1**	**3**			
	Medium	**2**	**3**			
	High	**3**	**3**		**1**	
**F02. Years of experience <u>IN MANAGEMENT</u>**	Low	**3**	**3**	**1**	**1**	
	Medium	**2**	**3**			
	High	**1**	**3**			
**F03. Education of the FLNM**	Máster	**1**	**-2**			
	Other management training	**1**	**-2**			
	Soft skills	**1**	**-2**			
	Digital competencies	**1**	**-2**			
	Nursing metodology	**1**	**-2**			
**F04. Leadership style**	Transformac.	**2**	**1**			
	Transac.	**1**	**1**			
	Other style	**0**	**1**			

ORGANISATION RELATED ITEMS.	Level	Points	Weight	Administrative support	clinical co-leader support	second FLNM support
**O01. Digitalisation and technological support for HR and HRM management. Information systems.**	Low	**3**	**3**	**1**	**2**	
	Medium	**2**	**3**			
	High	**1**	**3**			
**O02. Evaluation of competencies and detection of training needs of the unit's professionals.**	Low	**1**	**2**			
	Medium	**2**	**2**		**1**	
	High	**3**	**2**		**2**	
**O03. Participation in research projects, EBP (Evidence Based Practice), R&D&I (Research, Development and Innovation) and dissemination of results.**	Low	**1**	**2**			
	Medium	**2**	**2**		**1**	
	High	**3**	**2**		**2**	
**O04. Existence of trainees for tutoring in the unit / service.**	Low	**1**	**2**			
	Medium	**2**	**2**			
	High	**3**	**2**		**1**	
**O05. Existence of cross-cutting units, commissions or working groups that provide support to the FLNM.**	Low	**1**	**2**			
	Medium	**2**	**2**			
	High	**3**	**2**		**1**	
**O06. Performance of on-call duties as nurse in charge**	Low	**0**	**2**			
	Medium	**1**	**2**			

4. INTERPRETATION OF RESULTS

Score: The maximum possible score is 225 points

SOC < 50 % (111 points or less)	The span of control is below the normal limit. There is potential for growth
SOC > 50% (112 points) and < 60% (135 points)	The span of control is within appropriate values
SOC >60%.	The span of control is excessive. The FLNM requires support.

Clarifications on the type of support indicator:

• A calculation has been established taking into account the score of all items with respect to each recommended type of support.

• Values above 50% indicate the advisability of considering the need for that type of support for the FLNM.

• In case several types of support are recommended for the same FLNM, the one that best suits the needs, as well as the situation and objectives of the organisation, should be evaluated."
